# Recent Data on Cellular Component Turnover: Focus on Adaptations to Physical Exercise

**DOI:** 10.3390/cells8060542

**Published:** 2019-06-05

**Authors:** Anthony MJ Sanchez, Robin Candau, Henri Bernardi

**Affiliations:** 1 Laboratoire Européen Performance Santé Altitude, EA4604, University of Perpignan Via Domitia, Faculty of Sports Sciences, F-66120 Font-Romeu, France; 2 Université de Montpellier, INRA, UMR866 Dynamique Musculaire et Métabolisme, F-34060 Montpellier, France; robin.candau@umontpellier.fr; 3 INRA, UMR866 Dynamique Musculaire et Métabolisme, F-34060 Montpellier, France; henri.bernardi@inra.fr

**Keywords:** autophagy, mitophagy, mitochondria, exercise, AMPK, FOXO, MTOR, parkin

## Abstract

Significant progress has expanded our knowledge of the signaling pathways coordinating muscle protein turnover during various conditions including exercise. In this manuscript, the multiple mechanisms that govern the turnover of cellular components are reviewed, and their overall roles in adaptations to exercise training are discussed. Recent studies have highlighted the central role of the energy sensor (AMP)-activated protein kinase (AMPK), forkhead box class O subfamily protein (FOXO) transcription factors and the kinase mechanistic (or mammalian) target of rapamycin complex (MTOR) in the regulation of autophagy for organelle maintenance during exercise. A new cellular trafficking involving the lysosome was also revealed for full activation of MTOR and protein synthesis during recovery. Other emerging candidates have been found to be relevant in organelle turnover, especially Parkin and the mitochondrial E3 ubiquitin protein ligase (Mul1) pathways for mitochondrial turnover, and the glycerolipids diacylglycerol (DAG) for protein translation and FOXO regulation. Recent experiments with autophagy and mitophagy flux assessment have also provided important insights concerning mitochondrial turnover during ageing and chronic exercise. However, data in humans are often controversial and further investigations are needed to clarify the involvement of autophagy in exercise performed with additional stresses, such as hypoxia, and to understand the influence of exercise modality. Improving our knowledge of these pathways should help develop therapeutic ways to counteract muscle disorders in pathological conditions.

## 1. Introduction

Skeletal muscles are fundamental to the body’s maintenance, and disorders in their function or metabolism are related to numerous diseases. Improved skeletal muscle activity has a significant effect on major processes in the body, such as the regulation of glucose homeostasis, contributing to enhanced health. Importantly, our capacity to recover from illness also depends on skeletal muscle oxidative capacity. Hence, skeletal muscle displays noteworthy adaptive responses from several stimuli, such as contractile activity, nutritional interventions, and environmental factors like hypoxia. These conditions may induce a transitory cellular stress leading to numerous adaptations, such as modifications in fiber composition, improvements of cell ability to renew cellular proteins and organelles, and modifications of muscle size [1,2,3].

Among the molecular sensors involved in adaptations to training, the adenosine monophosphate (AMP)-activated protein kinase (AMPK) is an enzyme composed of two regulatory domains (i.e., AMPK-ß, AMPK-γ) and a catalytic domain (i.e., AMPK-α). AMPK is a critical enzyme for preserving cellular homeostasis under conditions of low energy [4,5]. AMPK activity is increased by several energy stresses, including hypoxia/ischemia [6,7], electrical-stimulated muscle contraction [8,9], starvation [10], and physical exercise [11,12,13]. When cellular ATP is depleted, AMP modulates AMPK activity in an allosteric way, thereby promoting the phosphorylation of a threonine residue (Thr-172) within the α subunit by other enzymes called the “AMPK kinases” (AMPKK) [14]. There are three AMPKK proposed to date, the Ca^2+^/calmodulin- dependent protein kinase ß (CaMKKß) [15,16], the liver kinase B1 (LKB1) [17,18], and the transforming growth factor ß-activated kinase 1 (TAK-1) [19]. Of note, the binding of ADP, like AMP, prevents AMPK Thr-172 dephosphorylation [20]. On the contrary, AMPK is inhibited by ATP and glycogen [21,22]. AMPK is involved in cell metabolism and several data have highlighted the physiological relevance of its activation in skeletal muscle [4,23]. Thus, AMPK promotes energy production through the anaerobic and aerobic systems (i.e., glycolysis and oxidation of fatty acids) and, conversely, inhibits glycogenesis and cholesterol synthesis [5,24,25,26,27,28]. AMPK enhances mitochondrial biogenesis by stimulating PGC-1α (peroxisome proliferator-activated receptor gamma coactivator 1 alpha) expression [29]. A study by Jager et al. also showed that AMPK phosphorylates PGC-1α on two residues (Thr-117 and Ser-538) in vitro and in cells [30]. PGC-1α consecutively regulates the activity of PPARs (peroxisome proliferator-activated receptors) and NRFs (nuclear respiratory factors), leading to mitochondrial adaptations [30,31,32].

AMPK’s biological functions are not limited to energy metabolism. In the last decade, AMPK was found to coordinate cell component turnover. AMPK decreases protein translation by reducing the activity of the mechanistic (or mammalian) target of rapamycin complex 1 (MTORC1) signaling, and promotes protein breakdown by regulating several component of the ubiquitin-proteasome and autophagosome-lysosome systems [5]. Major targets of AMPK are the forkhead box class O subfamily proteins 1 and 3 (FOXO1 and FOXO3, respectively). FOXO proteins are important transcription factors highly conserved through evolution and their various functions in skeletal muscle (i.e., cell cycle, DNA damage repair, apoptosis, energy metabolism, and oxidative stress resistance) have been recently reviewed [33]. In recent years, the AMPK-FOXO3 axis has been extensively studied with an important focus on processes regulating organelle turnover, especially mitophagy.

In this review, recent discoveries on AMPK-MTORC1 and AMPK-FOXO axes in the coordination of muscle organelle renewal and the importance of physical exercise on both acute and chronic adaptations are discussed. The multiple modes of regulation of these sensors are detailed, as their implication in the regulation of skeletal muscle protein and organelle turnover, especially mitophagy. Apparent discrepancies between the data are discussed in regard to the methodology used to access autophagy or mitophagy activity. The functions of newly identified actors in protein and organelle quality control, specifically the diacylglycerol kinase ζ (DGKζ), Parkin (RING-between-RING E3 ligase), and Mul1 (mitochondrial E3 ubiquitin protein ligase), are also presented. We finally discuss the impact of exercise modality, hypoxia, and examine the current limitations in the literature to suggest other perspectives.

## 2. AMPK and MTORC1 Pathways

### 2.1. AMPK/MTORC1 Axis in Organelle Quality Control during Exercise

**Figure 1:**
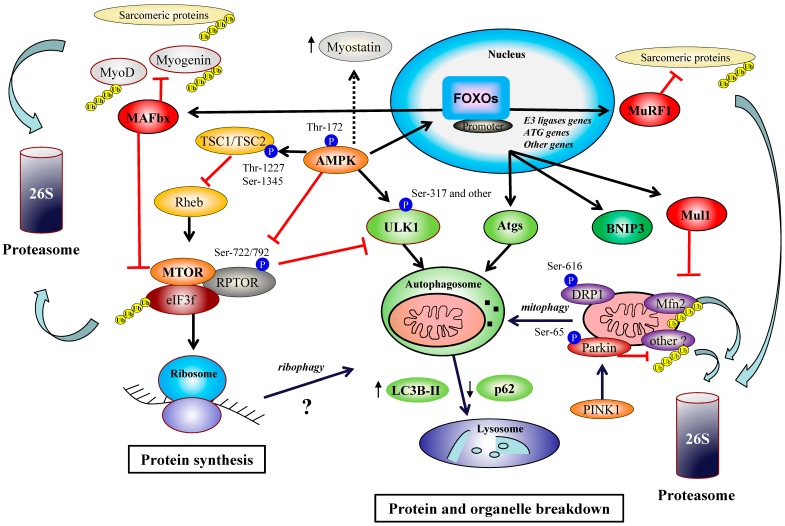
(AMP)-activated protein kinase (AMPK), forkhead box class O subfamily protein (FOXO), and mechanistic (or mammalian) target of rapamycin complex 1 (MTORC1) in the regulation of protein and organelle turnover. FOXO proteins increase the transcription of the E3 ubiquitin protein ligases muscle atrophy F-box (MAFbx)/atrogin-1, muscle RING finger 1 (MuRF1), mitochondrial E3 ubiquitin protein ligase (Mul1), several autophagic genes (Atgs), and BCL2/adenovirus E1B 19 kDa protein-interacting protein 3 (BNIP3) in muscle cells. MTOR and AMPK differentially modulate autophagy initiation by phosphorylation of the Unc-51-like kinase (ULK1). AMPK also activates FOXO1 and FOXO3 and tuberous sclerosis complex 2 (TSC)1/TSC2 complex, and inhibits MTORC1 complex through phosphorylation of the associated regulatory protein of MTOR complex-1 (RPTOR). MAFbx/atrogin-1 and MuRF1 target sarcomeric proteins. MAFbx/atrogin-1 also targets factors involved in cell growth including the transcription factors Myogenin and MyoD, and the eukaryotic initiation factor 3f (eIF3f). PTEN-induced putative kinase protein 1 (PINK1)/Parkin and Mul1 axes enhance mitophagy through ubiquitination of mitochondrial proteins.

Protein synthesis machinery is globally decreased during exercise. The MTORC1 pathway is a central regulator of protein turnover under conditions of increased external loading by the regulation of ribosomal translation. Thus, the kinase MTOR modulates mRNA translation and protein synthesis by regulating major regulators of ribosomal activity, 4E-BP1 (eukaryotic translation initiation factor 4E-binding protein 1) and S6K1 (ribosomal protein S6 kinase 1). MTOR phosphorylates S6K1 at Thr-389 (its hydrophobic motif), which, in turn, phosphorylates translational effectors such as rpS6 (ribosomal protein S6) and eIF4B (eukaryotic translation initiation factor 4B) [34,35]. In addition, PDK1 (phosphoinositide-dependent kinase-1) phosphorylates S6K1 at Thr-229 provides its full activation [36,37]. 4E-BP1 phosphorylation by MTOR on Thr-37/46 provides its disconnection from the preinitiation complex (PIC) promoting the transcription of protein-coding genes [38,39]. In a study conducted in mice, moderate endurance exercise decreased the phosphorylation state of MTORC1 signaling (i.e., MTOR, S6K1, rpS6, and 4E-BP1) from 90 min [40]. These modulations were concomitant with a raise of the phosphorylation state of eiF2α (Ser-51), an indicator of endoplasmic reticulum stress and AMPK activation. The AMPK mediates inhibition of MTORC1 through phosphorylation of MTOR at Thr-2446 [41], of the tuberous sclerosis complex 2 (TSC2) at Thr-1227 and Ser-1345 [42] and the associated regulatory protein of MTOR complex-1 (RPTOR) on two well-conserved serine residues (Ser-722/792) [43,44] leads to the sequestration of the raptor by 14-3-3 proteins [44]. These events promote the inhibition of Rheb (Ras homolog enriched in brain) and overall MTORC1 inhibition (Figure 1). However, data on the involvement of AMPK/RPTOR axis for MTORC1 inhibition during endurance exercise are lacking.

A single bout of exercise may lead to a strong stimulation of muscle protein synthesis during the recovery period. Synthesis of myofibrillar proteins is elevated after resistance exercise [45,46,47] and seems to become more pronounced in trained athletes compared to untrained individuals [48]. Endurance training also increases protein synthesis during the recovery since the MTORC1 pathway is altered in response to this type of exercise. Indeed, increased phosphorylation of several MTOR targets occur in both mice and humans after moderate and exhaustive endurance exercises [40,49,50]. However, selective activation of MTORC1 by specific intracellular signaling pathways is involved according to the exercise modality. Acute sprint exercise or electrical stimulation at high frequency increases PKB (protein kinase B or Akt) and MTOR phosphorylation during recovery [51,52]. Importantly, a recent study conducted in rats compared different resistance exercise models and suggested that hypertrophic response is correlated with the phosphorylation level of MTOR and its regulators or targets (i.e., Akt, the extracellular signal-regulated kinases ERKs, p38, the mitogen-activated protein kinases MAPKs, 4E-BP1) [53]. Finally, the study by Ogasawara highlighted that both rapamycin-sensitive and rapamycin-insensitive MTOR signaling regulate MTOR-dependent muscle protein synthesis during resistance exercise [54]. Concerning endurance exercise, several studies failed to observe modulation of Akt during recovery, whatever exercise intensity [52,55]. Thus, it has been proposed that an AMPK-Akt switch may be involved in the occurrence of specific adaptations to resistance and endurance training [55], with a more specific contribution of Akt/TSC1/TSC2 cascade for resistance, strength, and sprint exercises. This also means that Akt is not essential for MTORC1 activation and protein synthesis during endurance exercise. AMPK activation also contributes to decrease the rate of protein production during resistance exercise. However, protein translation augments from 1 h post-exercise even if AMPK activity becomes less pronounced from 2 h [56]. Finally, in individuals accustomed to training, MTORC1 signaling appears preferably involved for hypertrophy-inducing exercises and the AMPK axis seems to be more specific to endurance training adaptations [57]. It is interesting to note that a combination of endurance and resistance training may affect protein synthesis differentially. Indeed, AMPK activation via endurance exercise may negatively affect MTORC1 activation induced by resistance training. This effect is increased when the endurance exercise is carried-out after a bout of resistance exercise [58].

### 2.2. MTORC1 Regulators and Exercise: Recent Data on DGKs, FOXO, eIF3f and Cellular Trafficking

Among MTORC1 regulators, the PI3K (phosphoinositide 3-kinase)/Akt axis is notably modulated by exogenous nutrients and the release of growth factors [59,60,61,62]. MTOR phosphorylation during mechanical overload, and at the early phase of recovery, is related to MEK/ERK signaling through phosphorylation of TSC2 at Ser-664 but not to PI3K/Akt signaling [63]. ERK1/2 also regulates nuclear transcriptional factors, such as Elk-1 (E-26-like protein 1), c-Myc, c-Jun, and c-Fos that play a role in muscle growth [64]. Activation of ERK1/2 and MTORC1 seems to be required for full stimulation of protein synthesis in humans [65]. However, none of these MTORC1 regulators are recognized as critical actors for fiber hypertrophy during resistance exercise [59,60,61].

It is also recognized that mechanical stimulus enhances the activation of PLD (phospholipase D) and production of PA (phosphatidic acid), promoting activation of MTORC1. PA is a lipid messenger that binds to MTOR’s FKBP12- rapamycin binding domain, favoring its activation [61,66,67,68]. Nonetheless, PLD activity modulation does not seem only implicated in PA production or MTORC1 activation [69]. Thus, studies from Hornberger’s lab investigated the role of diacylglycerol (DAG) and DAG kinases (DGKs) in PA production during mechanical stimulation. DGKζ (DAG zeta) is known to be involved in PA accumulation via DAG phosphorylation and was strongly suggested to be essential for PA production and enhanced MTORC1 activity during mechanical stimulation. More recently, the same group explored the importance of DGKζ in muscle adaptations in vivo with a hypertrophic model of mechanical loading [70]. Thus, the authors reported that DGKζ isoform is the most highly raised and is of importance for muscle growth and hypertrophy. Studies on the mechanisms underlying these adaptations highlighted that DGKζ also suppresses FOXO3 activity, leading to a decline of MAFbx (muscle atrophy F-box)/atrogin-1 and MuRF1 (muscle RING finger 1) induction. Conversely, the expression of these E3 ligases during exercise was increased in DGKζ knock-out muscles, confirming the role of DGKζ in limiting protein breakdown during mechanical overload. Even if the precise mechanism needs to be identified, DGKζ relocation in the nucleus seems to be essential for inhibiting FOXO3 transcriptional activity during mechanical overload. Interestingly, the authors also found that mechanical overload increases expression of the eukaryotic initiation factor 3 subunit f (eIF3f), and this increase is totally blunted in DGKζ knock-out muscles. Given the importance of eIF3f in protein translation process, it can be hypothesized that DGKζ-dependent alteration of FOXO pathway during mechanical overload may also have effects on the translational machinery. Indeed, the FOXO-dependent E3 ligase MAFbx/atrogin-1 is well known to target eIF3f leading to its proteasomal degradation [71].

The role of eIF3f and MTORC1 intracellular trafficking in adaptation to exercise has been also recently investigated. eIF3f belongs to the translation initiation factor complex eIF3f among its 13 subunits involved in mRNA translation initiation. In the last two decades, studies from Leibovitch’s lab highlighted eIF3f’s involvement in skeletal muscle protein synthesis and hypertrophy [72]. It was demonstrated that a TOS (TOR signaling) motif in eIF3f operates as a scaffold to connect MTORC1 with its translational substrates and to support the initiation of cap-dependent translation [73]. Starvation muscle atrophy is suppressed when an eIF3f mutant insensitive to polyubiquitination by MAFbx is overexpressed, showing its critical role in muscle homeostasis during such a stress [74]. More recently, eIF3f was found to be essential for mouse embryonic development and its partial depletion reduces adult skeletal mass and amplifies muscle loss during disuse by mainly modulating protein synthesis [75]. Concerning exercise, an increase of eIF3f expression was evidenced during overload [70], suggesting a role in adaptations to exercise. Studies in human tissue model that used immunofluorescence approaches identified a type of cellular trafficking involving eIF3f that occurs during a single bout of resistance exercise [76]. Thus, the complex composed of MTORC1/eIF3f was found to co-localize with the lysosome, where the GTPase Rheb is known to trigger the kinase activity of MTOR enhancing MTORC1 substrates phosphorylation [77]. In accordance, studies in vitro and in animals highlighted that MTORC1 recruitment to the lysosome surface is critical to raise MTOR kinase activity [78,79]. After resistance exercise, the MTORC1/LAMP2 complex was found to rapidly translocate at the cell membrane in close proximity to capillaries [76]. Importantly, TSC2 abundance at the cell membrane was also reduced with a dissociation from Rheb, suggesting a decrease of MTOR inhibition favorable to its full activation. These innovative results suggest that, at least in humans, MTORC1 is recruited and activated at the cell periphery following resistance exercise. Interestingly, a protein–carbohydrate beverage post-exercise does not alter MTORC1/eIF3f translocation but increases the interaction between MTOR and eIF3f [76], an association well recognized to drive MTORC1 activation and enhance MTOR target activity [72]. Thus, a bout of resistance exercise may enhance mRNA translational capacity through the association and the translocation to the cell periphery of MTOR and its positive regulators eIF3f and Rheb.

**Figure 2:**
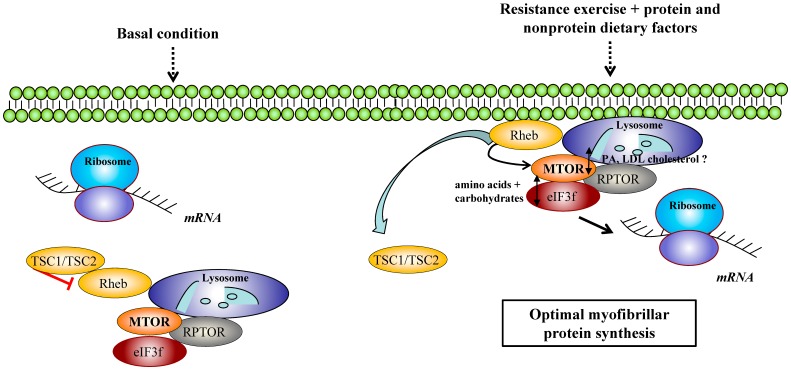
Cellular trafficking of mechanistic (or mammalian) target of rapamycin complex 1 (MTORC1) and lysosome after resistance exercise. In response to resistance exercise, MTORC1 co-localizes with the lysosome and translocates at the cell membrane. MTORC1 recruitment to the lysosome surface is critical to increase MTOR kinase activity. Contractions lead to a decrease of tuberous sclerosis complex 2 (TSC2) abundance and, conversely, to the activation of MTOR by the GTPase Ras homolog enriched in brain (Rheb). A protein-carbohydrate beverage post-exercise increases the interaction between MTOR and eukaryotic initiation factor 3f (eIF3f) without altering MTORC1/eIF3f translocation. Whole eggs ingestion enhances MTORC1 co-localization to the lysosome after resistance exercise.

Interestingly, nonprotein dietary factors also influence post-exercise myofibrillar protein synthesis. In humans, it was recently reported that the ingestion of egg whites alone results in lower myofibrillar protein synthesis activation than the ingestion of whole eggs during the recovery from resistance exercise [80]. In accordance, whole egg ingestion increases the phosphorylation level of MTOR, 4E-BP1, rpS6 to a greater extent than egg white ingestion. Whole egg ingestion was also found to increase MTORC1 co-localization with the lysosome after resistance exercise, and this result was correlated with higher rates of myofibrillar protein synthesis [81]. This observation suggests a better mRNA translational capacity after whole egg consumption than after egg white consumption. The underlying mechanisms have been partially studied and PA was proposed to have a role among the potential factors involved in MTORC1 recruitment to the lysosome. Egg yolk contains phosphatidylcholine and oleic acid that can be converted to PA via de novo synthesis, and DAG [82,83,84]. In addition, the egg yolk is enriched in low-density lipoprotein (LDL)-derived cholesterol that was shown to play a role in MTORC1 recruitment to the lysosome in a SLC38A9-Niemann-Pick C1 (a sterol transport system) signaling complex fashion [85]. In summary, these results highlighted the importance of a new intracellular trafficking mechanism and nonprotein dietary factors that drive optimal myofibrillar protein synthesis after resistance exercise (Figure 2).

## 3. AMPK and FOXO Transcription Factors

### 3.1. FOXO Homologues in Energy Metabolism and Post-Translational Modifications

Four FOXO members (FOXO1/3/4/6) are expressed in muscle. FOXO6 represses PGC-1α expression and low intensity exercise reduces FOXO6 induction [86], suggesting that exercise-induced PGC-1α may be partially dependent on FOXO6. Nonetheless, data concerning this factor remain limited in this tissue. FOXO1, 3, and 4 have been more extensively investigated, notably due to their important roles in cell cycle, apoptosis, muscle growth, and muscle regeneration [33]. FOXO3 regulates MyoD (an essential myogenic differentiation factor) transcription [87]. FOXO1 and FOXO3 play critical roles in energy homeostasis by favoring mitochondrial metabolism, inhibiting glycolysis, and enhancing lipolytic flux [88,89,90,91]. FOXO3 plays a role in the regulation of mitochondrial genome through AMPK and the mitochondrial Sirtuin 3 (SIRT3) [92,93]. FOXO1 and FOXO3 also regulate exercise-induced angiogenesis. FOXOs contribute to the repression of muscle angiogenic response through the induction of thrombospondin 1 (THBS1) during the first days of chronic exercise. Importantly, FOXO repression is critical for long term adaptations to endurance training, especially for angiogenesis [94,95].

**Figure 3:**
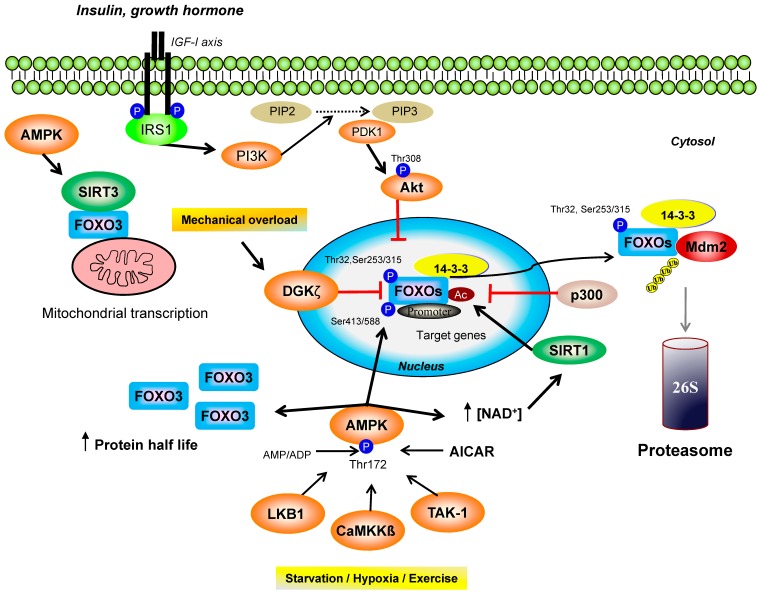
(AMP)-activated protein kinase AMPK and forkhead box class O subfamily protein FOXO regulation in skeletal muscle. FOXO proteins are phosphorylated and inhibited by protein kinase B (PKB or Akt) in response to insulin or growth factor. Under the condition of energy stress, AMPK, which is activated by the AMPK kinases (AMPKK) Ca^2+^/calmodulin- dependent protein kinase ß (CaMKKß), liver kinase B1 (LKB1), and transforming growth factor ß-activated kinase 1 (TAK-1), phosphorylates and increases FOXO3 activity. AMPK is also involved in FOXO deacetylation through Sirtuin 1 (SIRT1) and Sirtuin 3 (SIRT3). AMPK activation by 5-aminoimidazole-4-carboxamide-1-ß-D-ribofuranoside (AICAR) stabilizes FOXO3 by increasing its protein half-life. Moreover, AMPK favors the association between FOXO3 and mitochondrial DNA through SIRT3, mediating the transcription of mitochondrial genes. During mechanical overload, FOXO3 transcriptional activity is inhibited by diacylglycerol kinase ζ (DGKζ) by a mechanism independent of DGKζ kinase activity within the nucleus. Cytosolic FOXO3 is targeted by E3 ligase murine double minute 2 (Mdm2) for degradation via the ubiquitin-proteasome system. FOXO3 and FOXO4 are acetylated by the histone acetyltransferase p300. Abbreviations undefined in the main text: IRS1, insulin receptor substrate 1; PIP2, phosphatidylinositol 4,5-bisphosphate; PIP3, phosphatidylinositol 3,4,5-triphosphate.

FOXO proteins are regulated by several post-translational mechanisms, and numerous enzymes have been identified as kinases of FOXO, including AMPK, Akt, and, more recently, DGKζ. In muscle cells, AMPK activates FOXO3 by phosphorylation at Ser413/588 [23,96]. We recently found that AMPK activation by 5-aminoimidazole-4-carboxamide-1-ß-d-ribofuranoside (AICAR) extends the FOXO3 protein half-life in skeletal muscle primary cells [97]. AMPK also increases the cellular NAD^+^ level, thus enhancing the activity of histone deacetylase Sirtuin 1 (SIRT1) [98]. This process induces the deacetylation of FOXO1 and 3 leading to their activation [98]. Conversely, Akt, when inhibited by the phosphatidylinositol 3-kinase (PI3K), is responsible for FOXO1 protein translocation from the nucleus to the cytoplasm, leading to its inactivation [99]. Akt phosphorylates FOXO3 on several residues (Thr-32, Ser-253/315), and the phosphorylation of residues Thr-32 and Ser-253 promotes its cytoplasmic retention by a mechanism involving the 14-3-3 chaperone protein [100]. FOXO regulation by Akt induces a decrease in the binding between FOXO and its DNA sequences targets and, consequently, decreases FOXO transcriptional activity [101,102]. In addition, FOXO3 is targeted by the histone acetyl-transferase p300 for its ubiquitination by Mdm2 (E3 ligase murine double minute 2) and subsequent degradation by the proteasome [103]. In muscle, p300 alters differentially the expression of FOXO 1 and 4 without affecting the expression of FOXO3 [104]. Furthermore, p300 alters FOXO3 and FOXO4 activity but increases the nuclear localization of FOXO1 and the transcription of FOXO1-dependant genes underlining the differential regulation of the FOXO homologues [104]. Finally, DGKζ has been recently found to suppress FOXO3 activity in skeletal muscle [70], extending the role of the DGK to the regulation of catabolism (Figure 3).

### 3.2. AMPK/FOXO Axis in Organelle Quality Control during Exercise

In addition to its role in muscle metabolism, AMPK/FOXO axis represents a major actor in cellular components turnover via the ubiquitin-proteasome and autophagosome-lysosome proteolytic systems. The ubiquitin-proteasome pathway involves E3 ubiquitin-ligases that target substrates to the 26S proteasome for degradation after poly-ubiquitination. FOXO1 and FOXO3 regulate the transcription of crucial ubiquitin ligases, including MuRF1, Trim32 (tripartite motif-containing protein 32), and MAFbx/atrogin-1, implicated in myofibrillar protein removal (i.e., myosin light chain 1 and 2, myosin heavy chain protein, myosin-binding protein C, actin, tropomyosin, troponins, alpha-actinin, desmin, Z-bands, thin filaments), and myogenic/growth factors (i.e., MyoD, myogenin, eIF3f) [71,89,99,105,106,107,108,109,110,111,112,113,114,115,116]. Autophagy is a critical stress response that allows the replacement of protein, organelles, and other cellular components. In the first stage, proteins or other cellular constituents (e.g., mitochondria, ribosomes, peroxisomes, endoplasmic reticulum, lipids, polysaccharides) are incorporated in a double-membrane vesicle called the autophagosome. Then, the content of the autophagosome is removed by another vesicle named the lysosome that contents acid hydrolases. FOXO3 promotes the transcription of a plethora of autophagy genes (ATGs) involved in autophagosome biogenesis and maturation, including ATG4B, ATG12L, Beclin, BNIP3 (BCL2/adenovirus E1B 19 kDa protein-interacting protein 3), GABARAPL1 (GABA Type A Receptor Associated Protein Like 1), LC3 (microtubule-associated protein light chain 3), PI3KIII, ULK2 (Unc-51-like kinase) [106,117]. Of note, BNIP3/BNIP3L are involved in mitophagy since BNIP3 is a mitochondrial receptor that directly connects to the Atg8 homolog LC3 and GABARAP, leading to the recruitment of autophagosome to damaged mitochondria [118,119,120]. FOXO1 also activates the lysosomal protease cathepsin L in skeletal muscle [121]. In mice, several autophagy markers are more expressed in slow-twitch muscles but basal autophagy flux appears to be higher in glycolytic muscles, suggesting that autophagy in glycolytic muscle might be more tightly regulated [122]. Importantly, AMPK and MTOR differentially regulate the initiator of autophagy ULK1 (Atg1) through phosphorylation [123,124,125], noticeably in skeletal muscle [5,23,126]. AMPK phosphorylates ULK1 at several residues (Ser-317/467/555/777) leading to autophagy initiation in condition of energy stress. Conversely, MTOR inhibits ULK1 by phosphorylating Ser-757 when nutrients are plentiful.

In the last decade, studies have revealed that acute endurance exercise affect the expression and phosphorylation level of markers involved in protein and organelle removal. FOXO1 and FOXO3 level increases after exhaustive exercises [33], and it was found that several proteolytic actors (i.e., MAFbx/atrogin-1, MuRF1, LC3B-II, and Atg12 expression, etc.) and proteasome β2 subunit activity may be enhanced after marathon or ultra-endurance exercise [127,128]. Some of these modulations were also reported in response to exercise performed at moderate intensity [129,130,131]. Increases in protein breakdown may be useful to favor cell component turnover during recovery, or, alternatively, amino acids can serve as substrates when exercise is prolonged. Interestingly, the enhanced muscle protein anabolic response with ingestion of essential amino acids and carbohydrates during the recovery of resistance exercise seems primarily due to an increase in protein translation compared to modulation of protein degradation [132]. More conventional endurance exercise may augment the expression of autophagy markers according to exercise intensity and the nutritional state. When performed in a fasted state, exercise promotes more important increases of autophagy markers (LC3B, GABARAPL1 lipidation, cATG12 protein level, p62 mRNA level) and more specific inductions of actors involved in mitophagy (BNIP3 and Parkin expression) compared to exercise conducted in a fed state [133]. Consistently, rises in DRP1 (dynamin related protein 1) phosphorylation, a GTPase involved in fission of mitochondria, quickly occurs during exercise, including endurance exercise conducted at moderate intensity (40–50% of $\dot{V}O_{2}max$) in sedentary rodents [40]. Exercise at moderate intensity quickly increases phosphorylation of AMPK and induces initiation of the autophagy pathway through ULK1 [40]. An increase in autophagy markers (i.e., LC3 lipidation and p62 expression) can be observed near to exhaustion [40]. Importantly, the modulation of autophagy markers during endurance exercise differs between rodent and human muscles. Indeed, regarding LC3 lipidation or p62 expression, acute endurance exercise seems to promote enhanced autophagosome content in mice, while the opposite can be found in humans [134,135]. However, 60 min of cycling exercise at moderate intensity increases ULK1 phosphorylation concomitantly to a decrease in LC3 lipidation in human skeletal muscle, suggesting an initiation of autophagy [129]. It will be necessary to define if these contradictive results found in humans can be explained by a decrease in autophagy or alternatively by a fast degradation of the autophagosome by the lysosome because of an abrupt induction of overall system. Development of valid markers of autophagic flux in humans should contribute to a better understanding of this pathway.

Autophagy is an essential system for muscle maintenance since chronic autophagy deficiency leads to increased proportion of centralized nuclei and pro-apoptotic markers, reduced force, altered twitch kinetics in glycolytic muscle, as well as enhanced calpain and proteasomal enzymatic activity [122]. In cardiac and skeletal muscles, autophagy plays a role in the exercise-related metabolic effects [130,131]. Disruption in autophagy decreases endurance performance and alters glucose metabolism. In addition, autophagy failure results in mitochondria degeneration, sarcoplasmic reticulum distension and disorganization of sarcomere [136], confirming the critical role of autophagy in mitochondria and myofiber homeostasis. In response to chronic exercise, autophagy appears to be needed for exercise training-induced mitochondrial remodeling and fiber-type transition [137].

### 3.3. Exercise and Autophagy/Mitophagy Flux

The majority of the aforementioned studies investigated autophagy at the transcriptional or translational level. However, these approaches are not suitable for the interpretation of autophagy activity. The “autophagic flux assay”, an accurate method to access autophagy activity, is based on the turnover of LC3 and p62. Inhibitors of autophagy (colchicine, chloroquine, NH_4_Cl, or bafilomycin A1) are used to block the incorporation of the autophagosomes into the lysosome or to reduce the activity of lysosomal enzymes. This method avoids incorrect analysis of LC3-II or p62 protein level. For example, LC3-II content can be increased when autophagy activity is enhanced but also when the latter stages of the process (e.g., fusion between autophagosome and lysosome) is altered. Per contra, a decrease of LC3-II levels could mean either a decrease or, when lysosomal degradation is fast, an elevation in autophagy activity. Experiments with autophagy flux are lacking, especially in the evaluation of the responses to chronic exercise in vivo. However, in a recent investigation [138], the authors used colchicine treatment to establish a link between autophagy modulation during endurance training and mitochondrial biogenesis in mice skeletal muscle. Thus, autophagy suppression by colchicine abrogated mitochondrial adaptations linked to training. More recently, studies from Hood’s lab examined the impact of ageing and chronic contractile activity on basal muscle autophagy and mitophagy flux. In these experiments, mitophagy flux was assessed on isolated mitochondria. The authors found that aged muscles present accelerated basal mitophagy flux, especially in intermyofibrillar mitochondria [139,140]. This result indicates that, during ageing, autophagy activity is increased to promote the targeting of damaged organelles, especially mitochondria. Importantly, chronic contractile activity decreases mitophagy flux in both aged and young muscles [139,140]. Hence, one might hypothesize that, in this context, chronic contractile activity improves mitochondria quality and decreases the necessity to recycle them through the autophagosome-lysosome pathway. Additional investigation using mitophagy flux experiments is needed to examine the effect of exercise training on basal mitochondria turnover in vivo. Importantly, the same group also reported that contractile activity may normalize autophagy flux and reverse mitochondrial abnormalities during autophagy suppression [141]. In that respect, exercise may represent an effective therapeutic issue to counterbalance diseases with defects in organelle replacement. However, to date, no data have been made available on the effect of exercise on the turnover of other organelles such as ribosomes or endoplasmic reticulum that represent crucial components in muscle protein homeostasis.

## 4. The Emerging Roles of Parkin and FOXO3-Dependant Mul1 Pathway in Organelle Turnover and Adaptations to Exercise

In the last decades, the E3-ubiquitin ligase Parkin has been implicated in the control of mitophagy with a particular focus on neuronal degeneration, especially during Parkinson disease (PD) [142,143,144]. PINK1 (PTEN- induced putative kinase protein 1) is a mitochondrial serine/threonine protein kinase that is activated by the depolarization of mitochondrial membrane. PINK1 phosphorylates and enhances Parkin activity [144]. Data highlighted that Parkin phosphorylation at Ser-65 is necessary for its mitochondrial translocation, leading to the degradation of several actors involved in mitochondrial dynamics and motility [142,145]. Parkin is known to ubiquitinate TOMM20 (translocase of outer mitochondrial membrane 20), the mitochondrial fusion protein Mitofusin 1/2 (Mfn2 1/2), DRP1, Fis1, Miro, and VDAC (voltage-dependent anion channel) [146,147,148,149,150,151], leading to the translocation of autophagy receptors to mitochondria (i.e., LC3, SQSTM1/p62 and NBR1/autophagy cargo receptor) [152] (Figure 1). In addition to its roles in mitophagy and mitochondrial dynamics, Parkin targets the transcriptional repressor of PGC-1α PARIS (ZNF746, zinc finger protein 746), favoring mitochondrial biogenesis [153]. Finally, a role of Parkin in the production of mitochondrial-derived vesicles playing a role in mitochondrial quality control has also been revealed [154,155,156,157,158].

A recent study investigated the involvement of Parkin in the muscle phenotype of PD [159]. The authors reported that the mitochondrial uncoupler carbonyl cyanide m-chlorophenylhydrazone (CCCP) promotes PINK1/Parkin-mediated mitophagy in C2C12 cells, and myotube atrophy. Ablation of Parkin resulted in accumulation of dysfunctional mitochondria and myotubular atrophy, suggesting that Parkin plays a role in skeletal muscle mitochondrial removal. More recently, Gouspillou and co-workers highlighted, for the first time, the critical role of Parkin in contractile and mitochondrial properties of healthy muscle [160]. The authors found that Parkin ablation causes muscle contractile dysfunction associated with higher cross-sectional area of type-IIb fibers. Importantly, Parkin ablation results in mitochondrial dysfunction with reduced maximal mitochondrial respiration and mitochondrial uncoupling but without alteration of mitochondrial content. This study has also suggested that Parkin ablation favors oxidative stress, decreases mitochondrial fusion, and increases mitochondrial fission. Even if specific mitophagy flux has not been assessed, the authors also found an increase of autophagy markers in Parkin^−/−^ muscles. Thus, these data are consistent with global mitochondria alteration and Parkin appears to be a critical actor in maintaining contractile properties efficiency in normal skeletal muscle. In addition, a recent study from Hood’s lab [161] revealed that Parkin and mitophagy are of importance for training-induced mitochondrial adaptations. However, in this study, the authors showed that Parkin ablation does not significantly affect basal mitophagy flux. In response to a single bout of endurance exercise, enhanced mitophagy flux and alterations of PGC-1α signaling were observed in Parkin deficient mice, but mitochondrial content was increased in a similar extension to the wild-type population. This result indicates that Parkin/PGC-1α axis seems unessential for mitochondrial biogenesis during exercise. Importantly, the authors demonstrated that acute endurance exercise elevates mitophagy flux and training attenuates this elevation in wild-type but not in Parkin deficient animals. However, training does not alter basal mitophagy flux, even if the presence of Parkin to mitochondrial membrane is enhanced in the basal state. These data seem to indicate that a lower rate of mitophagy may occur thanks to training adaptations that may lead to a better mitochondria quality. In agreement, the authors also found that training increased mitochondrial content in Parkin knock-out mice, but these mitochondria showed several dysfunctions.

The E3-ligase Mul1 (or MULAN/GIDE/MAPL) was recently proposed to be associated to the AMPK-FOXO3 signaling pathway in muscle [97]. Mul1 plays a role in the control of mitochondrial quality by coordinating mitochondrial removal and dynamics but also apoptosis [162,163,164]. Mul1 is upregulated by AMPK activation in primary myotubes [97] and AMPK could block the preservative effects of IGF-1 on contractility of sensory-innervated muscle cells through Mul1 enhancement [165]. In skeletal muscle, changes in Mul1 expression lead to the degradation of Mfn2 and mitochondrial fission, inducing mitochondrial elimination [166]. Mul1 also favors the fragmentation of mitochondria through DRP1 stabilization [167]. Interestingly, Mul1 was also found to stabilize ULK1 in HeLa cells [163]. However, these recent findings have not yet been evaluated in muscle cells. Even if these data highlight a role of Mul1 in mitochondrial turnover and muscle atrophy, data on exercise are still limited and there is a need to examine the implication of Mul1 in adaptations to training. A recent study revealed that Mul1 and the mitophagy pathway seems not to be involved in adaptations to training in muscle of patients with type 2 diabetes [168]. However, an increase of Mul1 protein expression, but not mRNA level, has been observed during acute endurance exercise in the muscles of healthy rodents [40], indicating a potential role for Mul1 during such a stress.

## 5. Exercise in Hypoxia

Athletes currently use training under hypoxia as a method to enhance performance at sea level or to prepare competitions at altitude. The addition of hypoxia during training elicits higher metabolic stress and can promote selective adaptive responses for aerobic performance [169,170]. Recently, it was found that repeated-sprint training in hypoxia also enhances repeated sprint ability in swimming and team-sports [171,172,173]. Another protocol in which high-intensity exercises were conducted under hypoxia and recoveries in normoxia, was also proposed for highly-trained athletes to provide additional effects on endurance performance [174]. The most recognized effects of training under hypoxia on skeletal muscle are related to oxidative capacity, capillary density, mitochondrial density, and enhanced blood glucose utilization [170,175,176]. However, occurrence of adaptations depends on several factors (e.g., hypoxic dose and duration) [177], and studies also reported no further positive effect on performance at sea level [178,179,180].

Acute exposition to normobaric hypoxia was found to modulate basal protein turnover markers. However, the majority of the works on hypoxia mainly focused on the modulation of protein translation markers [181,182,183,184] and data on the effect of exercise conducted under hypoxia on protein balance are lacking. Nevertheless, studies from Deldique’s lab recently suggested that acute exposition to hypoxia increases or does not have effect on the MTORC1 pathway [185,186]. This discrepancy between studies could be explained by differences in the nutritional pattern that affect insulin (or catecholamines) concentration. Indeed, high plasma insulin concentration may upregulate the MTORC1 pathway during exposure to hypoxia [185]. Concerning autophagy, recent studies from Masschlelein and co-workers investigated its regulation during acute normobaric hypoxia in humans [186]. The authors observed a raise in the LC3-II/I ratio and a reduction of p62 expression at rest, suggesting enhanced autophagy. In addition, moderate cycling exercise increased the BNIP3 mRNA level, a marker of mitophagy [186]. However, no study has performed autophagy flux measurement in response to both acute and chronic hypoxia, limiting possible interpretations. Finally, concerning resistance training in humans, a recent study from Deldique’s lab [187] highlighted that hypoxia (FiO_2_: 14%) blunts the activation of protein synthesis and down-regulates the transcriptional program of autophagy. Importantly, resistance exercise performed in hypoxia seems to initiate the transcription of genes involved in satellite cell incorporation that potentially participate in gains of force production observed in the long term [187]. Additional studies are also required to evaluate the gains in muscle mass and force production compared to normoxia.

Although studies on the acute effects of hypoxia on protein turnover are emerging, chronic adaptations to altitude or normobaric hypoxic training remain largely unexplored. It is documented that chronic hypoxia can cause skeletal muscle atrophy in rodent through a downregulation of protein translation and enhanced proteolysis, as well as alterations in oxidative metabolism [188,189,190]. In humans, high altitude hypoxia exposure (4559 m) for 7–9 days induces a decline of iron transport-related protein expression, tricarboxylic acid cycle, oxidative phosphorylation, and oxidative stress [191]. Moreover, MTOR level may be reduced in such a condition [191], suggesting alteration in protein translation. However, the effects of ambient hypoxia on both others proteins synthesis markers (i.e., translational initiation markers) and catabolic signaling pathways (i.e., autophagy) remain to be clarified in humans, especially in combination with exercise training and nutritional strategies [185,192].

## 6. Muscle Contraction Regimens and Cell Component Turnover

Numerous investigations have shown that resistance training with eccentric actions performed at high intensities may have higher benefits for muscle strength and hypertrophy compared to concentric or isometric contraction modes [193]. In addition, early adaptations have been reported with eccentric overload training [194]. Protein turnover pathways can be affected by different modes of contraction and the higher effects of eccentric mode on muscle growth seems to be associated with a greater activation of MTORC1 pathway [195]. However, contraction mode appears less influential on muscle hypertrophy with prolonged high-volume resistance training and protein and carbohydrate supplementation becomes more critical factors to further increase muscle mass [195]. Furthermore, when the magnitude of the force–time integral is normalized, studies in both rodents and humans found that eccentric mode induces similar anabolic responses to concentric mode [196,197]. From a molecular viewpoint, MTORC1 activation after eccentric contractions seems more related to PA synthesis than the PI3K/Akt signaling pathway [198]. Even if muscular hypertrophy involves the MTORC1 pathway during the post-exercise period [199], many weeks of training do not necessarily induce major changes on the basal MTORC1 pathway [195]. Under the same force–time integral, with regard to proteolytic markers (i.e., FOXO3 and ULK1 phosphorylation, LC3B-II/I ratio, and MAFbx/atrogin-1, MuRF1 and p62 expression), the study from Ato and co-workers suggests that the contraction mode does not appear as a factor that may differentially regulate proteolytic pathways in the early phase of muscle contractions [200]. During exercise, a study from Sandri’s lab highlighted that autophagy seems important to preserve mitochondrial function during damaging muscle eccentric contractions [201]. Altogether, these data still suggest that the magnitude of force–time integral should be the main factor to explain differences in anabolic or catabolic response. The energy cost of eccentric actions is lower compared with concentric contractions, and eccentric exercise allows one to develop higher loads during a training session. In addition, autophagy flux experiments remain to be performed to avoid misinterpretation of the modulation of autophagy or mitophagy according to contraction mode. Thus, from our point of view, further studies have to consider these features.

Some studies explored the response to the combination of eccentric endurance exercise and hypoxia in skeletal muscle. Thus, the study from Klarod and co-workers suggests that downhill walking performed at a low altitude may present some advantages for physical fitness in pre-diabetic men [202]. In this study, eccentric endurance exercise training at a low altitude (from 850 to 1360 m) was shown to improve aerobic performance. At a moderate altitude (from 2000 to 2447 m), the same exercise training program increased the biological antioxidant activity of plasma. In rodents, the study from Rizo-Roca investigated if intermittent hypobaric hypoxia combined with aerobic exercise may be beneficial for recovery from eccentric-damaging exercises. The authors found that this strategy may reverse the signs of muscle damages and reinforce or preserve the fiber oxidative phenotype in response to several weeks of training [203]. The same group reported that this strategy may improve important mitochondrial aspects (i.e., fission/fusion markers and the expression of actors involved in mitochondrial biogenesis) during recovery from eccentric exercises [204]. These results open important perspectives on the use of hypoxia combined with aerobic exercise as a recovery method from damaging exercises in both athletes and patients. However, to provide further recommendations and better understand cellular adaptations, more studies have to be developed, especially studies on myofibrillar protein synthesis and mitochondria remodeling, in response to the eccentric exercise performed in hypoxia.

## 7. Conclusions and Perspectives

The preservation of muscle mass and oxidative capacity are essential for maintaining quality of life. In the past few years, advances have expanded our understanding on the impact of exercise on the events that govern organelle turnover and the role played by crucial factors, especially AMPK, MTORC1, and FOXO. Concerning protein synthesis, a major discovery of this last decade is that DGKζ appears as a key regulator of MTORC1 during overload. Importantly, the modulation and trafficking of eIF3f and its potential regulation through DGKζ/FOXO3 axis has been, for the first time, pointed out in the context of exercise. In addition, our knowledge on the physiological significance of MTORC1 recruitment to the lysosome and to the cell periphery has been significantly improved by using immunofluorescence approaches. Concerning protein breakdown during exercise, Parkin and Mul1 have been recognized as critical E3-ligases for normal skeletal muscle and mitochondria maintenance. Parkin appears essential for mitochondrial adaptations to endurance training and the maintenance of functioning mitochondria. However, the potential role of Mul1 in mitochondrial adaptations to chronic exercise has to be addressed. Furthermore, studies on hypoxia highlighted that hypoxic stress may blunt the MTORC1 pathway after resistance training but compensative mechanisms (i.e., transcription of satellite cells regulators) are potentially involved in hypertrophy and strength gains. Studies have to be initiated, in particular, with measurements of autophagy/mitophagy flux to reinforce our knowledge on the effects of training in hypoxia on organelle quality control. Regarding the mode of contraction, eccentric exercises lead to a greater activation of the MTORC1 pathway, probably through a PA-dependent mechanism. Finally, further studies on this topic and the impact of nutritional interventions have to be developed, to better understand skeletal muscle adaptations to training, thanks to organelle quality, and to develop countermeasures during illness.
